# Predictors of fall risk in older adults using the G-STRIDE inertial sensor: an observational multicenter case–control study

**DOI:** 10.1186/s12877-023-04379-y

**Published:** 2023-11-13

**Authors:** Marta Neira Álvarez, Cristina Rodríguez-Sánchez, Elisabet Huertas-Hoyas, Guillermo García-Villamil-Neira, Maria Teresa Espinoza-Cerda, Laura Pérez-Delgado, Elena Reina-Robles, Irene Bartolomé Martin, Antonio J. del-Ama, Luisa Ruiz-Ruiz, Antonio R. Jiménez-Ruiz

**Affiliations:** 1 Department of Geriatrics, Foundation for Research and Biomedical Innovation of the Infanta Sofía University Hospital and Henares University Hospital, (FIIB HUIS HHEN), European University, 28702 Madrid, Spain; 2 School of Experimental Sciences and Technology, Rey Juan Carlos University, 28933 Madrid, Spain; 3 Physical Therapy, Occupational Therapy, Rehabilitation and Physical Medicine Department, Rey Juan Carlos University, 28922 Madrid, Spain; 4 Centre for Automation and Robotics, UPM‐CSIC, 28500 Madrid, Spain; 5 Department of Geriatrics, Foundation for Research and Biomedical Innovation of the Getafe University Hospital, 28905 Madrid, Spain; 6 Department of Geriatrics, Gastón Baquero Residential Centre, 28108 Alcobendas Madrid, Spain; 7 Department of Geriatrics, Torrelaguna Residential Centre, 28180 Torrelaguna, Spain; 8 Department of Geriatrics, Guadalajara University Hospital, 19002 Guadalajara, Spain; 9 Universidad Alcalá (UAH), Madrid, Spain

**Keywords:** Elderly, Falls, Frailty, Gait analysis, IMU, Telemedicine

## Abstract

**Background:**

There are a lot of tools to use for fall assessment, but there is not yet one that predicts the risk of falls in the elderly. This study aims to evaluate the use of the G-STRIDE prototype in the analysis of fall risk, defining the cut-off points to predict the risk of falling and developing a predictive model that allows discriminating between subjects with and without fall risks and those at risk of future falls.

**Methods:**

An observational, multicenter case–control study was conducted with older people coming from two different public hospitals and three different nursing homes. We gathered clinical variables ( Short Physical Performance Battery (SPPB), Standardized Frailty Criteria, Speed 4 m walk, Falls Efficacy Scale-International (FES-I), Time-Up Go Test, and Global Deterioration Scale (GDS)) and measured gait kinematics using an inertial measure unit (IMU). We performed a logistic regression model using a training set of observations (70% of the participants) to predict the probability of falls.

**Results:**

A total of 163 participants were included, 86 people with gait and balance disorders or falls and 77 without falls; 67,8% were females, with a mean age of 82,63 ± 6,01 years. G-STRIDE made it possible to measure gait parameters under normal living conditions. There are 46 cut-off values of conventional clinical parameters and those estimated with the G-STRIDE solution. A logistic regression mixed model, with four conventional and 2 kinematic variables allows us to identify people at risk of falls showing good predictive value with AUC of 77,6% (sensitivity 0,773 y specificity 0,780). In addition, we could predict the fallers in the test group (30% observations not in the model) with similar performance to conventional methods.

**Conclusions:**

The G-STRIDE IMU device allows to predict the risk of falls using a mixed model with an accuracy of 0,776 with similar performance to conventional model. This approach allows better precision, low cost and less infrastructures for an early intervention and prevention of future falls.

## Introduction

Population aging is the result of successful health and social policies, with those over 65 being the group that has had the most growth in recent decades. But, as the aging population increases, more individuals will be at risk of developing chronic diseases, disability, and dependence.

Falls are one of the most important geriatric syndromes and one of the main causes of disability; they occur at all ages but specially over 65 years when frailty, sarcopenia and other multiple causes are more prevalent.

According to the World Health Organisation (WHO), 684,000 people die every year due to falls the second cause of accidental death in the world, in addition to conditioning important functional consequences. The three highest risk groups are children, workers, and the aged population. However, the elderly are the group with the highest risk of complications so recognizes falls as a public health problem of the first magnitude [1].

Significant complications in the elderly accompany falls; psychological impact, which can condition new falls and secondary functional impairment, physical consequences such as soft tissue injuries, rarhabdomyolysisr head trauma that occurs in 10% of cases, 5% suffer fractures and in 1–2% have a hip fracture that is the one with the greatest functional impact, mortality and hospital costs. Secondarily these complications condition institutionalization, functional and quality of life loss and direct and indirect health costs [2].

Falls typically arise from the complex interplay of various factors rather than a singular cause. Intrinsic factors, such as age, sex, and chronic conditions like diabetes, dementia, or Parkinson's disease, in combination with the effects of medication and environmental hazards, can disrupt balance and impact an older individual's postural responses, thereby heightening the risk of falls. This risk is particularly pronounced in specific circumstances, such as during transfers or while navigating challenging terrain [3].

In young individuals, falls result from external situations like sports or working activities. Still, in the older adult, a minimal external factor can lead to falling by combining multiple intrinsic and extrinsic factors. The multifactorial falls risk assessment allows identifying all these factors in order to develop individualised, tailored fall prevention plans. The identification of the subjects at risk of suffering from falls is crucial since it allows to act in specially susceptible populations and therefore reduce the incidence and prevalence of falls [4]. As well as improving their quality of life and their participation in the community.

However, to date, screening tools to detect risk subjects have shown variable results [5]. and some of them with application exclusively in certain settings (community, acute units, surgical, rehabilitation or residences) [6–8].

Finally, there are few studies in which new diagnostic tools such as posturography mechanical sensors or inertial sensors are incorporated. However, their use has emerged as an approach of great interest since they allow greater precision and richness of data, in some cases with lightweight sensors of easy use, portable and with low cost [9–12].

Recently, the results of a study that evaluates the applicability of the G-STRIDE electronic device based on inertial sensors, in evaluating subjects with and without falls have been published. The results showed that the device detects spatio-temporal gait parameters accurately, and were capable of discriminate between subjects with and without falls. Furthermore, significant correlations between the gait parameters and the functional tests commonly used were found [13]. Since the precision and discriminative capacity of G-STRIDE are promising, a relevant question remained to be answered: what is the predictive performance of G-STRIDE to predict fall probability.

Therefore the objective of this study is to evaluate the use of the G-STRIDE prototype for predicting fall risk, defining the cut-off points that allow predicting fall risk, and to develop a predictive model that allows discriminating between subjects with and without falls while identifying those at risk of future falls.

## Methodology

This is an observational, multicenter case–control study in older adults with and without fall risk. The Research Ethics Committee approved the sstudy of the Hospital Universitario de la Paz (Registration Number: PI-4486).

The estimated effect size for a t-test for differences between two independent means based on a statistical power of 0.8 and an alpha error of 0.05 with an effect size of 0.8, a sample size of 84 subjects was estimated.

Participants were included from the out-patient clinic in two general public hospitals and three nursing homes from September 2021 to March 2022.

We adopted the World Health Organisation definition for falls [1]: “a fall is an event that results in a person coming to rest inadvertently on the ground or floor or other lower level”. According to this definition, we define the “Fallers Group” as those adults over 70 years who had one of the next circumstances:

- One fall with consequences in the last year (requiring medical attention)
- Two or more falls in the same period
- Gait and balance disorder
- Fear of falling or post-fall syndrome.

These criteria were based on those proposed by the American Geriatrics Society (AGS) and the British Geriatrics Society (BGS) to identify those patients with higher risk of falls who should be offered a multifactorial assessment [14].

The participants without falls were volunteers over 70 years that gave informed consent.

Exclusion criteria for the study were terminal illness with a life expectancy of fewer than six months.

### Clinical assessment

Clinical assessment was carried out in a single visit..The following datawere registered: sociodemographic characteristics, level of Physical Activity, Weight, Height, Body mass index (BMI), Deterioration Scale from Reisberg (GDS) [15], frailty assessment using the Standardised Frailty Criteria (FRG) [16], walking speed (Speed 4 m walk) [17], Short Physical Performance Battery Scale (SPPB) [18], Time up and go test (TUG) [19], andf fear of falling syndrome assessed using the Short Falls Efficacy Scale—International (Short FESI) [20].

### Gait analysis. The G-STRIDE system

**Fig.1:**
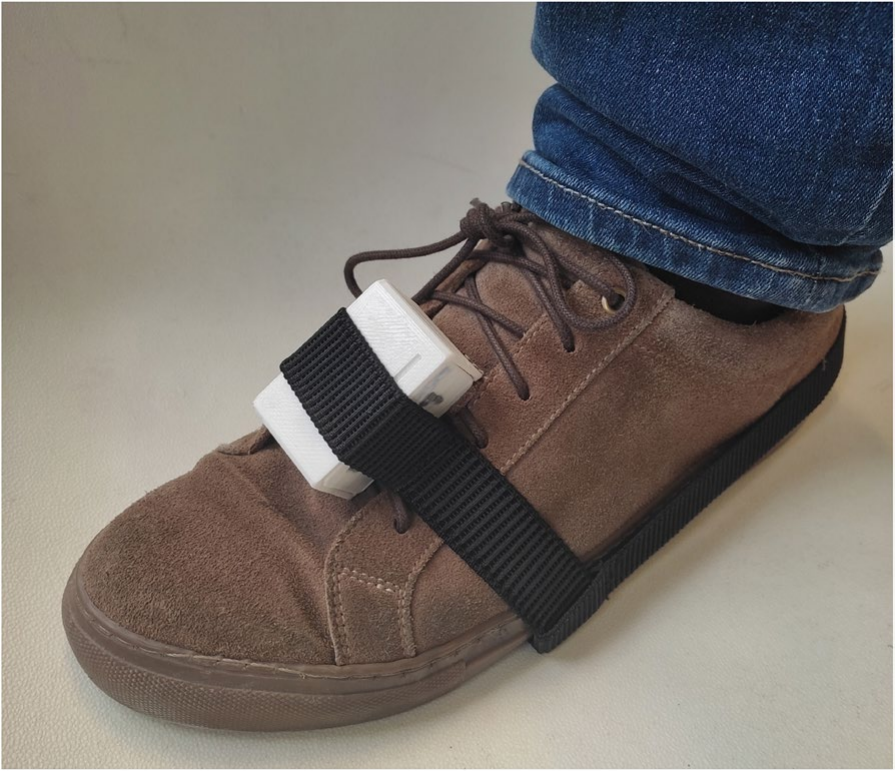
G-Stride IMU. Left: several units for tests. Right: IMU attached to a participant’s foot

Gait analysis was done using G-STRIDE system after the clinical assessment in the same visit. For the walking test, the device was placed on the top of the foot (Fig. 1) and after switching up the device, the participant was invited to walk freely for approximately 30 min. Participants from outpatient clinic walked around the hospital and those institutionalized walked around the nursing home. After this time, the data recorded was stored and the device was switched off.

The G-STRIDE device was presented in prevous paper [13]. It is comprised by an inertial sensor (IMU) and a processing electronics that allows obtaining kinematic-related variables (described below), store them in a SD card, and connect with a user interface. During the tests, no subject had any complications or problems derived from the use of the device.

The G-STRIDE is a lightweight device with dimensions 78 × 45x38 mm. It is comprised by an IMU and Arduino board that samples the data from the IMU during walking. It also features a Secure-Digital (SD) memory card to store the data from each test conducted, as well as Wi-Fi capacity to measure and visualize in real time walking data and system status. Besides, a Raspberry card is implemented to allow for off-line sensor data analysis stored in the SD card, and the execution of the inertial navigation zero-velocity-update (INS-ZUPT) algorithms to obtain the trajectory and orientation of the foot, and derive an all the walking-related variables defined by the clinicians to assess walking. These variables are then stored in a database hosted in the Raspberry itself and are post-processed. The G-STRIDE device was attached to the instep by an elastic band as shown in Fig. 1.

### Estimated gait parameters

**Fig. 2:**
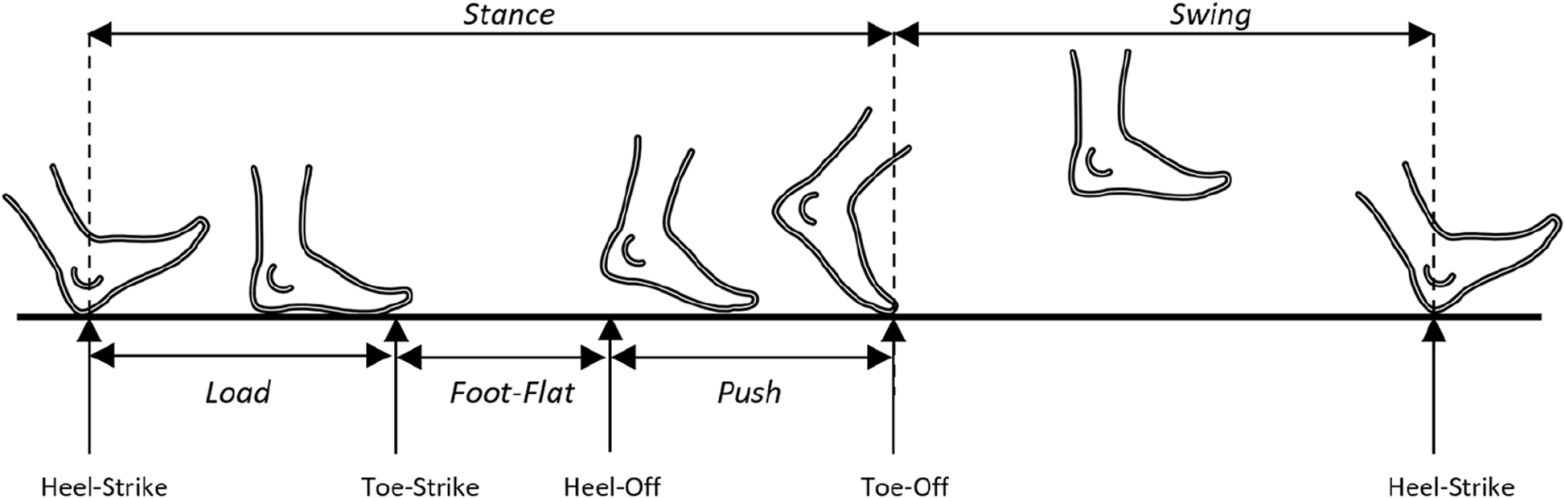
Diagram of gait phases (Stance, Swing, Load, Foot-Flat and Push) and events (Heel-Strike, Toe-Strike, Heel-Off and Toe-Off)
- Pitch angles at start and end of stance/swing, they are the angles that the foot forms with the ground during the heel-strike and toe-off events (see Fig. 3).
  - “Heel strike angle(deg)”: The maximum pitch angle at heel strike measured in degrees.
  - “Toe-off angle(deg)”: The maximum pitch angle at toe-off is measured in degrees.

The variables estimated by the G-STRIDE using the IMU on the foot of each participant are:

- “Total distance(m)”: The total distance traveled during long the free walk is measured in meters.
- “Total time(s)”: The total time taken in the long walk measured in seconds.
- “Total steps”: The total number of steps in the free walk.
- “Gait Cycle Time-GCT”: The mean Gait Cycle Time (GCT) measured in seconds. It is the time elapsed during a stride.
- “Velocity(m/s)”: The mean walking speed computed over the total detected steps measured in meters per second.
- “Cadence(steps/min)”: The mean cadence measured number of steps per minute.
- The time of each cycle in percentage (%) with respect to GCT. The phases and events are shown in Fig. 2:
  - “Swing time(% GCT)”: Swing time (from toe-off to heel strike) as percentage of GCT.
  - “Stance-Loading time(% GCT)”: From heel-strike to start of foot-flat time as percentage of GCT.
  - “Stance-FootFlat time(% GCT)”: Foot-flat (from start to end) time as percentage of GCT, it occurs between toe-strike and heel-off.
  - “Stance-Pushing time(% GCT)”: From heel-off to toe-off time as percentage of GCT.

**Fig. 3:**
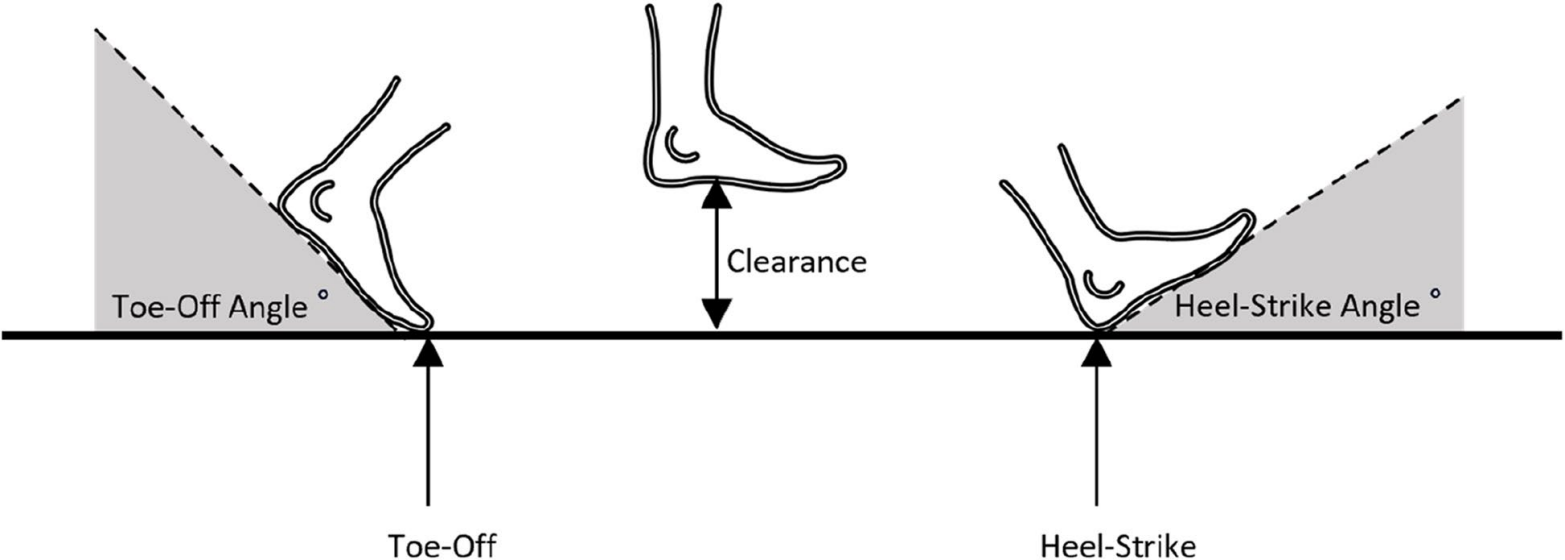
Diagram of foot angle during toe-off and heel-strike events, and foot clearance during swing phase
- “Clearance(m)”: The clearance or maximum height of the foot with respect to ground during the swing phase (see Fig. 3). It is obtained as the maximum value observed in Z.
- “Stride Length-SL(m)”: The Stride length (distance from one stance position to the next stance of the same foot) is measured in meters. It is the distance travelled during a stride (see Fig. 4).

**Fig. 4:**
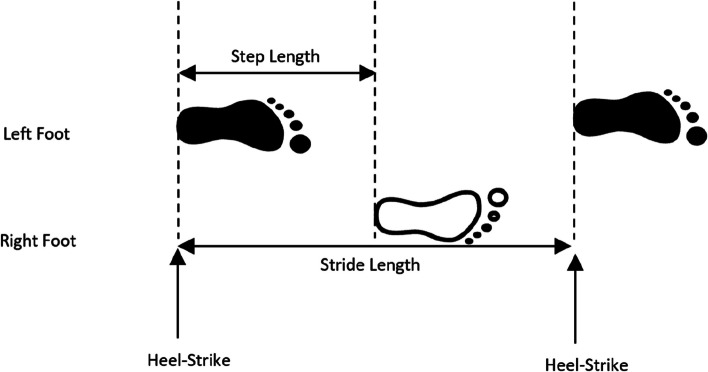
Diagram of gait spatial parameters: step and stride length
- “StepSpeed(m/s)”: The forward speed of foot only during the swing phase is measured in meters per second. It is calculated as the coefficient between Stride Length and Gait Cycle Time.
- “2D Path(m)”: The path length of the foot in the horizontal plane during a step (always equal or larger than SL), see Fig. 5. It is calculated as the position increment in XY.

**Fig. 5:**
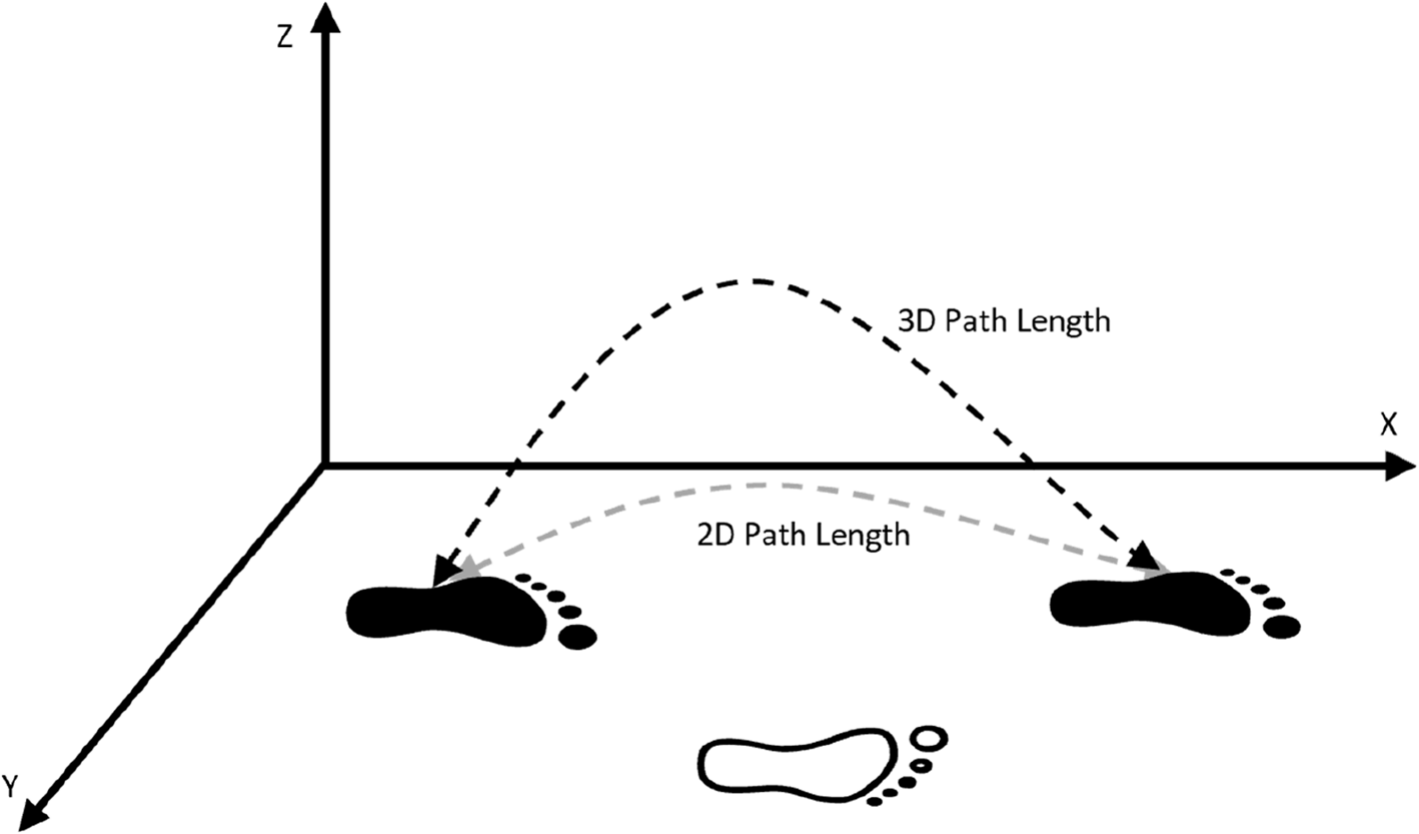
Diagram of 2D and 3D path length trajectory during a stride
- “3D Path(m)”: The path length of the foot in 3D space during a step (always equal to or larger than SL and 2D path), see Fig. 5. It calculated as the position increment in XYZ.

Additionally to the above mentioned parameters, which are computed as the mean on a step-by-step basics, we also estimate the standard deviation (STD) or variability among steps. These STD variables are also important to register the regularity or repetitiveness of walk, with higher STD indicating that the gait pattern is not too stable. The parameter estimation has been validated in [21], showing a stride-length mean relative error of below 4%.

### Statistical analysis

The sample complied with normality by Kolmogorov–Smirnov, so parametric tests were performed. We determined the demographic and anthropometric parameters as means and standard deviations for continuous variables (groups compared with the t student test) or percentages for the discrete variable (groups compared with Chi square test).

Statistical analysis was carried out with SPSS v.27 (Copyright© 2013 IBM SPSS Corp.) and the R language for statistical computing (R Foundation for Statistical Computing, Vienna, Austria) [22].

#### Logistic regression method for risk-of-fall cut-off and classification

As the FALLS variable is binary, we used a logistic regression learning in order to model and explain the variables that cause a fall, and to be able to predict future falls by classifying observations from new participants. This approach is used for cut-off point finding and also for muti-variable regression.

We used as training a cross-validation approach with 50 randomly generated subsets of all observations in our database. Each training subset contained 70% of the participants in the database. The remaining random subsets with 30% of the observations were used for testing the 50 generated models.

The confusion matrices and derived statistics were the average of all prediction results for all 50 fitted models, including the worst, the best and all other models in between (i.e. not using just the best-model results). The total number of testing observations were 2445 (50 × 0.3 × 163). This systematic methodology generates stable statistics (not changing with new iterations), so the accuracy values given in this paper are quite reliable of the expected performance.

The logistic regression models can incorporate demographic variables (such as age, gender, etc.) to address any potential imbalances in the sample distribution, particularly for variables like age or height, which directly influence walking speed.

Logistic regression is a valuable tool for categorical prediction, providing probability scores for observations. However, it has some limitations. When multiple co-linear variables are present, the stability of coefficients during convergence to a fit may be compromised. Logistic regression also struggles when dealing with a large feature space or a substantial number of categorical variables. Nonetheless, the regression models developed in this study exhibit reasonable performance with our dataset. It should be noted, however, that the addition of new variables to the models can lead to a decrease in performance. Therefore, there is a trade-off between model performance and the number of variables employed.

## Results

Table 1 shows basal characteristics of the study with 163 participants (86 of fallers group).

**Table 1:** Descriptive basal parameters

	Overall (*n* = 163)	Fallers Group (*n* = 86)	No fallers (*n* = 77)	t o X^2^
**Age** (min–max/ M ± DS)	70 – 98 / 82,63 ± 6,01	71 – 96 / 84,17 ± 5,48	70–98/80,9 ± 6,5	-3,44**
**Sex**				32,69***
Male (Fr (%))	45 (25,9)	19 (22,1)	26 (33,8)	
Female (Fr (%))	118 (67,8)	67 (77,9)	51 (66,2)	
**Weight** (min–max/ M ± DS)	33,1 – 105 / 64,28 ± 13,11	33,1 – 94,4 / 63,08 ± 13,44	37,6–105/65,62 ± 12,68	1,23
**Height** (min–max/ M ± DS)	1,32 – 1,84 /1,56 ± ,10	1,32 – 1,74 / 1,52 ± ,08	1,42–1,84/1,61 ± ,09	6,49***
**IMC** (min–max/ M ± DS)	,002 – 42,52 / 25,65 ± 6,10	,002 – 42,52 / 26,21 ± 7,45	16,46–35,49/25,02 ± 4,05	-1,27
**GDS** (min–max/ M ± DS)	1 – 7 /2,05 ± 1,6	1–7/ 2,35 ± 1,7	1–7/1,71 ± 1,43	-2,58 **
**Living_Site**				19,93***
Residence (Fr (%))	53 (30,5)	31 (36)	22 (28,6)	
Home (Fr (%))	110 (63,2)	55 (64)	55 (71,4)	
**Terrain**				13,92***
Mix (Fr (%))	53 (30,5)	9 (10,5)	44 (57,1)	
Flat (Fr (%))	99 (56,9)	71 (82,6)	28 (36,4)	
**Test_Site**				2,810
Hospital (Fr (%))	64 (36,8)	52 (60,5)	12 (15,6)	
Residence (Fr (%))	52 (29,9)	30 (34,9)	22 (28,6)	
Home (Fr (%))	47 (27)	4 (4,7)	43 (55,8)	

^**^p < 0.01

^***^p < 0.001; t Student; X^2^ Chi Square

Mean age was 82.6 ± 6.2 years, being older the group of fallers and 118 (72%) were women.

### Cut-off points and faller detection performance

Cut-off points are able to individually separate non fallers from group of fallers. A total of 46 cut-off values are presented in Tables 2 and 3. They were computed individually with a specific logistic regression for each of the variables. This list contains classical clinical parameters (Table 2) and those estimated with the footmounted IMU (G-STRIDE) (Table 3).

**Table 2:** Cut-off points for classical clinical parameters

	CutOff	intcpt	cofhim	with	*p*	itself
Time_4m_inalk	5.19	-2.53	0.48	4.66	0.0001	***
Speed_4m_walk	0.84	3.68	-4.33	-5.76	0.0001	***
SPPB_equilibrium	3.30	2.98	-0.90	-4.68	0.0001	***
SPPB_4mSpeed_score	3.21	3.10	-0.96	-5.20	0.0001	***
SPPB_ChairStand_score	2.79	1.14	-0.41	-3.19	0.001	**
SPPB_Total	9.03	3.41	-0.37	-5.13	0.0001	***
TUG	13.62	-2.71	0.19	5.02	0.0001	***
FES1	9.45	-2.72	0.28	4.54	0.0001	***

CutOff = It is a cutoff or limit value used in the analysis; intcpt = Refers to an intercept or constant in a regression model; cofhim: Represents a coefficient associated with a specific variable; with (Z): Indicates the relationship or association of a variable with respect to other variables or the outcome; *p*: It is the *p*-value, which is used to evaluate the statistical significance of the coefficients, *p* < 0.05; itself: Represents the value or outcome of a variable itself

**Table 3:** Cut-off points for IMU (G-STRIDE) parameters

	CutOff	intcpt	cofhim	with	*p*	itself
Total distance (m)	1028.713	2.214	-0.002	-5.40	0.0001	***
Total time (s)	1351.843	2.592	-0.002	-4.39	0.0001	***
Total steps	1135.392	2.791	-0.002	-5.01	0.0001	***
Gait Cycle Time—GCT (s)	1.205	-5.585	4.633	4.32	0.0001	***
GCT STD	0.094	-2.128	22.637	4.75	0.0001	***
stance—Pushing time (% GCT)	18.244	4.522	-0.248	-3.68	0.0001	***
stance—Pushing time STD	1.630	-0.752	0.462	1.69	0.09	
Swing (% GCT)	28.914	10.606	-0.367	-5.14	0.0001	***
Swing STD	2.257	-2.576	1.141	4.32	0.0001	***
Stance—Loading time (% GCT)	11.371	3.594	-0.316	-3.96	0.0001	***
Stance—Loading time STD	1.337	-0.431	0.322	0.88	0.376	
Stance—FootFlat time (% GCT)	41.860	-6.614	0.158	5.11	0.0001	***
Stance—FootFlat time STD	3.876	-1.862	0.480	3.71	0.001	***
Toe off angle (deg)	-53.855	4.308	0.080	5.18	0.0001	***
Toe off angle STD	-5.101	0.053	0.010	0.09	0.926	
Heel strike angle (deg)	15.595	2.344	-0.150	-4.94	0.0001	***
Heel strike angle STD	2.720	2.152	-0.791	-3.68	0.001	***
Cadence (steps/min)	50.492	5.941	-0.118	-4.34	0.0001	***
Cadence STD	3.846	-1.637	0.426	3.15	0.001	**
StepSpeed (m/s)	0.855	3.742	-4.376	-5.75	0.0001	***
StepSpeed STD	0.110	1.657	-15.064	-2.54	0.01	*
StrideLength—SL (m)	0.940	4.332	-4.611	-5.55	0.0001	***
StrideLength STD	0.120	1.151	-9.561	-1.93	0.05	
3D Path (m)	1.016	4.586	-4.515	-5.73	0.0001	***
3D Path STD	0.034	-0.055	1.614	0.27	0.789	
2D Path (m)	0.957	4.390	-4.586	-5.57	0.0001	***
2D Path STD	0.123	1.079	-8.790	-1.85	0.064	
Clearance (m)	0.180	1.010	-5.609	-3.61	0.001	***
Clearance STD	0.050	1.066	-21.450	-3.83	0.0001	***
Velocity (m/s)	0.784	3.650	-4.658	-5.64	0.0001	***

CutOff = It is a cutoff or limit value used in the analysis; intcpt = Refers to an intercept or constant in a regression model; cofhim: Represents a coefficient associated with a specific variable; with (Z): Indicates the relationship or association of a variable with respect to other variables or the outcome; *p*: It is the p-value, which is used to evaluate the statistical significance of the coefficients, *p* < 0.05; itself: Represents the value or outcome of a variable itself

The intercept and coefficients are also included in the table to let us know the direction of the effect. Negative coefficients mean that larger values in the variable causes lower probability of fall. On the contrary if coefficient is positive when a parameter increases so does the probability of fall.

As can be seen, most variables are significant.

The accuracy of fall risk estimation using just one cut-off is good but limited (68.5% for FES1, 68.9% for SPPB Total, 69.51% for SPPB equilibrium, 66.3% for FRG Total, 71.18% for ‘StrideLength-SL(m)‘or 72.4% for ‘StepSpeed (m/s)‘. Note the promising classification power (larger than 70%) of the last two G-STRIDE (IMU) variables. It is expected at least a slightly better performance when using several variables at the same time. This will be seen in next subsection using a multi-variable logistic regression approach.

Next, we present the different logistic regression models for three types of models: a) Using only conventional vavriables (clinical scales and variables), 2) G-STRIDE kinematic data alone, and 3) Mix model integrating the clinical scales and the kinematic variables obtained from the G-STRIDE.

### Logistic regression using clinical variables

Using the conventional variables, we fitted over the train data the logistic regression model represented in Table 4, where the first column represents the coefficients of the model. Most variables are as individuals discriminant (seen in the cut-off Table 2), but when combined with others appear less discriminant (e.g. SPPB total and”Speed 4 m walk” with *p* value greater than 0.9). For this combination of variables, the accuracy is 78.4% (Table 7), which is better than using any single cut-off classification (all lower than 70% as seen in last section).

**Table 4:** Logistic regression model using conventional variables

Characteristic	log(OR)^a^	95% CI^a^	*p*-value
FES1	0.14	-0.02, 0.32	**0.089**
FRG_Total	1.8	0.63, 3.2	** < 0.001**
TUG	0.04	-0.07, 0.17	0.5
GDS	0.00	-0.31, 0.31	> 0.9
Speed_4m_walk	-1.2	-4.3, 1.8	0.4
SPPB_Total	-0.04	-0.35, 0.27	0.8
Age	-0.02	-0.11, 0.08	0.8

^a^*OR* Odds Ratio, *CI* Confidence Interval

### Logistic regression using G-STRIDE kinematic variables

A selection of G-STRIDE kinematic variables was done using an iteractive process to exclude individual parameters that did not impacted in the performance of the model because of its correlation with other parameters. At the end we obtain a selection of parameters (including both means and standard deviation STD), those fitted in the logistic regression model represented in Table 5, where the first column represents the coefficients of the model. The most relevant variables are GCT STD, Stance-Foot Flat-time STD. The other variable appears to be less significant, but it is a dilution effect due to colinearity. The accuracy for this model is 68.0% (Table 7), which is not as good as expected. Remember that in the cut-off analysis a single parameter performed like this: 71.18% for ‘StrideLength-SL(m)‘ or 72.4% for ‘StepSpeed (m/s)‘ (Table 5). We estimate that could be possible to improve performance by reducing the number of terms, specially selecting the parameters that are easier to estimate (those with lower estimation error, below 5%) or by improving the estimation algorithms to make estimation more reliable in the most challenging parameters.

**Table 5:** Logistic regression model using IMU (G-STRIDE) variables

Characteristic	log(OR)^a^	95% CI^a^	*p*-value
Total distance (m)	0.00	0.00, 0.00	0.4
GCT ST	29	2.5, 59	**0.032**
StancePushing time (% GCT)	0.06	-0.40, 1.1	0.8
Swing (% GCT)	-0.11	-0.68, 1.0	0.8
Stance—Loading time (% GCT)	0.27	-0.36, 1.4	0.4
Stance—FootFlat time (% GCT)	-0.06	-0.52, 1.0	0.9
Stance—FootFlat time STD	-0.79	-1.5, -0.15	**0.015**
Toe off angle (deg)	0.04	-0.05, 0.13	0.4
Heel strike angle (deg)	-0.05	-0.24, 0.13	0.6
Heel strike angle STD	-0.56	-1.6, 0.38	0.2
Cadence (steps/min)	0.04	-0.22, 0.32	0.7
StepSpeed (m/s)	-2.8	-19, 12	0.7
StrideLength—SL (m)	0.52	-13, 15	> 0.9
Clearance (m)	4.1	-12, 23	0.6
Clearance STD	-13	-80, 46	0.7

^a^*OR* Odds Ratio, *CI* Confidence Interval

### Logistic regression using mixed clinical & kinematic variables

Finally, using a logistic regression mixed model, with 4 conventional & 2 G-STRIDE kinematic variables, we obtained the coefficients represented in Table 6. The most relevant variables are FES1, FRG Physical activity and StepSpeed. The accuracy is 77.6% (Table 7).

**Table 6:** Logistic regression model using mix variables

Characteristic	log(OR)^a^	95% CI^a^	*p*-value
FES1	0.24	0.08, 0.44	**0.002**
SPPB_equilibrium	-0.38	-1.0, 0.17	0.2
FRG_strength	-0.03	-0.12, 0.05	0.5
FRG_physical_activity	1.3	0.13, 2.6	**0.029**
StepSpeed (m/s)	-2.7	-5.4, -0.30	**0.027**
Stance—FootFlat time STD	-0.17	-0.60, 0.25	0.4

^a^*OR* Odds Ratio, *CI* Confidence Interval

**Table 7:** Statistics comparing the three models: Coventional (Conv model), G-STRIDE IMU (IMU model) and Mix model

Variable	Conv model	IMU model	Mix model	4 m walk model	SPPB model
Accuracy	0.78	0.69	0.77	0.74	0.69
Sensitivity	0.78	0.72	0.79	0.78	0.70
Specificity	0.79	0.64	0.75	0.64	0.75
PPV	0.80	0.69	0.78	0.74	0.70
NPV	0.77	0.68	0.76	0.74	0.68
Prevalence	0.51	0.54	0.52	0.55	0.52
F1score	0.79	0.71	0.78	0.76	0.70

### Comparing the three models: coventional, G-STRIDE IMU and mix regression models

**Fig. 6:**
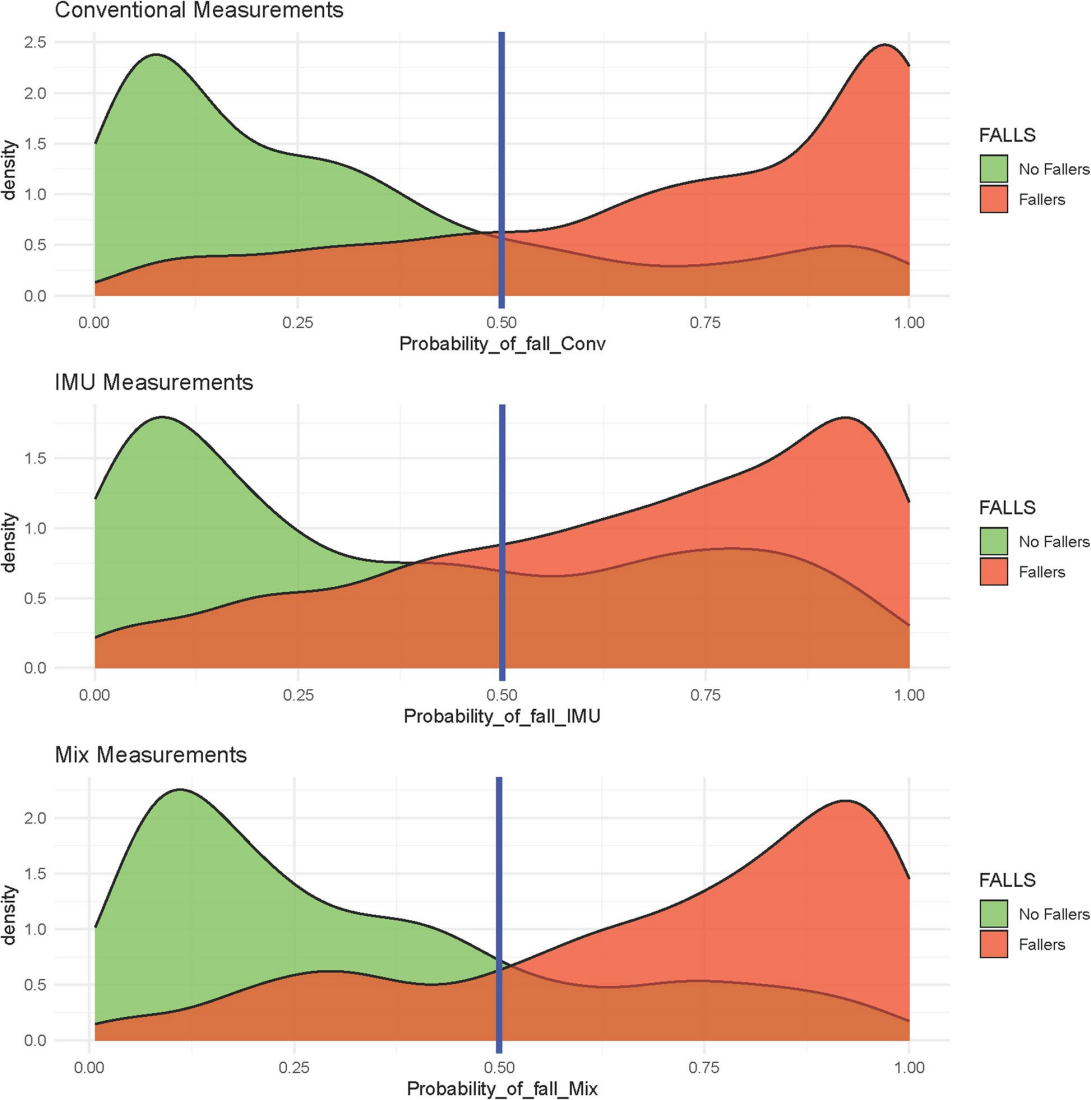
The capability for predicting the probability of fall for the three models (conventional model, IMU model and Mix model)

The capability for classifying or predicting the probability of fall, is shown in the form of histograms for an easy interpretation, in Fig. 2. The ideal 100% perfect classification will correspond to a full red histogram to the right of ther vertical cut-off line (100% true positives) and a full green histogram to the left of the vertical cut-off line (100% true negatives). However is evident the presence of some histogram tails that cross the cut-off line and represent the false positive or false negatives Fig. 6.

The confusion matrix derived for each of the 3 models (Conv, IMU and Mix) are presented in Table 7. The Mix model which includes a few parameters (6) is able to perform as well as a complete set of conventional study. It is also important to highlight the accuracy of other individual tests such as SPPB and the 4 m walk test (last two columns in Table 7) which are good, although show a lower performance when compared to the conventional and the mixed model that includes other complementary parameters.

## Discussion

The objective of this study was to compare the predictive performance of clinical parameters, obtained by conventional clinical evaluation and kinematic variables obtained by an electronic device based on inertial sensors (G-STRIDE) to identify fall risk in elderly subjects, defining cut-off points in the analyzed variables and regression models that allow predicting future fallers.

The results of the study show the cut-off points for risk of falls in both conventional clinical variables and the kinematic ones obtained by G-STRIDE. We investigated three regression models that allow identifying subjects at risk of future falls, with an accuracy > 0.784 (conventional clinical model), > 0.680 (model with G-STRIDE) and > 0.776 (mixed model).

We present 8 cut-off points for the clinical-functional variables assessed during the conventional evaluations for falls.In particular, it is important to note that the gait speed has the highest coefficient, demonstrating that the probability of falls increases as this parameter changes. Another essential aspect to mention is that the proposed cut-off point (0.849 m/sec) agrees with those mentioned in the literature [23, 24] In the case of other parameters such as SPPB, there is also agreement with what was previously published regarding the cut-off point that defines the risk of adverse events [18, 24]. In the case of another variable widely used in the assessment of falls, such as the TUG, the cut-off point coincides with that proposed with some authors [25] although other researchers suggest a higher cut-off point to detect future falls or disability [26, 27].Regarding the cut-off points in the parameters obtained by the device, it is the first approximation made of these characteristics and this will allow to know after the evaluation of the patient with the device that aspects of the gait are pathological and therefore, on which it should be possible to do tailored interventions, facilitating decision making. In addition, it can have a future application for the development of tools or app devices that facilitate the visualization of results, streamlining and simplifying the decision making of the clinician. Although there is an incrising number of studies using inertial sensors for gait analysis [11, 12] only some evaluate cut-off points, suggest specific analysis of stance sub-phases or improves TUG performance with “instrumented TUG” [28, 29]

Regarding predictive models that allow identifying subjects at risk of future fall we have studied three models and while the three models showed the similar results, the mixed model allow to have a more information of these clincial and biomechancial features related to falls, which provides a more comprehensive fall assessment. This screening tool for falls risk assessment could be used in both community and residential settings where the device has been evaluated.

Unfortunately, screening tools published to date have limited or insufficient ability to predict future falls [5, 30]. Several reviews address the analysis of different screening tools, and it appears that, in community settings, the TUG is the most widely recommended, with a sensitivity varying according to the studies between 0.68 and 0.76, a specificity between 0.49 and 0.74 and an AUC between 0.72 and 0.80 [26, 31, 32]. these differences may be due not only to the methodology used but also to the type of patients, or cut-off points chosen, and the differences are also observed in other tools so that authors recommend using several complementary tools [30, 32].

The mixed clinical-kinematic G-STRIDE model that shows an accuracy of 0,776. This approach to fall risk assessment is novel, since there are no predictive mixed models that sum the main clinical/exploratory risk factors with those collected by sensors in real word for falls prediction. We have found an interesting study by Martínez-Ramirez et al. that propose the use of a mixed model with trunk kinematic parameters during walking finding a more accurate frailty classification as the model could improve the early detection of prefrail status [33].

On the contrary, there are also a limited number of studies that explore the use of predictive models based on inertial systems or other sensory methodology and although there is a wide variety of technological solutions, they have been tested in different locations and with different measurements resulting in heterogeneous and insufficient results to reach firm conclusions [34].

In Fig. 2, it can be seen a good separability among fallers (red) and no fallers (green), using as a cut-off point the value 0.5. The output of the logistic regression indicates the probabilities, which if lower than 0.5 means a prediction as a”non faller”, and a probability larger than 0.5 is considered as”faller”. The tails within the wrong response (red tail to the left and green tail to the right) can be false positive or negative, or even could be the warning of future risk of fall for non-up today fallers, or no risk of fall for previous fallers. It is necesary to test these models in future studies to confirm the results.

This has strong implications, since the same results can be obtained from a reduced number of tests with complementary conventional measurements, such as the FES1, equilibrium, strength and the knowledge of physical activity by the patient; with the complement of some IMU-based features. In fact, the scientific literature identifies various measuring instruments as possible predictive tools of falls in the elderly [35, 36] but maintain the biases of human subjectivity or lack of precision details as the G-STRIDE device can offer.

The present study has several strengths such as sample size, advanced age representation, have been carried out in different settings (out-patient clinic, nursing homes and home) or collecting numerous functional tests. However, it is also necessary to point out that it would have been interesting to make a follow up to know clinical evolution of participants to detect future falls and evaluate the proposed predictive models. We believe that this could be the objective for a future study.

We can therefore conclude that the G-STRIDE IMU device allows to evaluate up to 17 gait parameters identifying 24 cut-off points and predict the risk of falls using a mixed model with an accuracy of 0,776.In this way, G-STRIDE IMU device contributes to improving falls evaluation in elderly in a more flexible and agile way, in real life conditions and with greater accuracy.

## Data Availability

Data availability at https://zenodo.org/record/6883292#.YtrAKnZByUk.
